# Packaging of alphavirus-based self-amplifying mRNA yields replication-competent virus through a mechanism of aberrant homologous RNA recombination

**DOI:** 10.1128/mbio.02494-24

**Published:** 2024-09-25

**Authors:** Tessy A. H. Hick, Corinne Geertsema, Reindert Nijland, Gorben P. Pijlman

**Affiliations:** 1 Wageningen University and Research, Laboratory of Virology, Wageningen, the Netherlands; 2 Wageningen University and Research, Marine Animal Ecology Group, Wageningen, the Netherlands; University of Colorado School of Medicine, Aurora, Colorado, USA

**Keywords:** self-amplifying mRNA, replicons, virus-like replicon particles, RNA recombination, replication-competent virus, aberrant homologous RNA recombination

## Abstract

Messenger (m)RNA has taken center stage in vaccine development, gene therapy, and cancer immunotherapy. A next-generation of mRNA is the self-amplifying (sa)mRNA, which induces broad and long-lasting immunity at a lower dose which provides better clinical outcomes in conjunction with fewer adverse effects. SamRNA, also known as “replicon” RNA, encodes the replication machinery of an alphavirus together with an antigen. Efficient delivery of replicon RNA to target tissues can be accomplished by packaging the replicon RNA in virus-like replicon particles (VRPs) via co-transfection of producer cells with defective helper RNA(s) encoding the alphavirus structural proteins. During the manufacture of VRPs, however, there is a potential risk of RNA recombination, which may lead to the formation of replication-competent virus (RCV). To investigate the factors influencing the unwanted RCV formation, we evaluated how sequence homology orchestrates alphavirus RNA recombination. Several combinations of complementing alphavirus replicon and helper RNAs varying in length of sequences overlap were co-transfected in mammalian cells. The culture fluid was serially passaged to detect RCV. Nanopore sequencing of cells after the first passage in combination with amplicon-based Sanger sequencing of RCV in the culture fluid after four passages led to the detection of RNA recombination. RCV was generated between replicon and helper RNAs with sequence homology in either the non-structural or structural genes, whereas RNAs without overlapping gene regions did not generate RCV. Remarkably, no sequence overlap was detected at the recombination junction sites in the RCV genome, suggesting a mechanism of “aberrant homologous RNA recombination.” Accordingly, we conclude that the alphavirus RNA recombination process leading to the formation of RCV is homology-assisted and can be prevented by avoiding sequence homology between replicon and helper RNAs.

**IMPORTANCE:**

There is a growing interest in the use of self-amplifying (sa)mRNA vectors for next-generation vaccine development, gene therapy, and cancer immunotherapy. The delivery of samRNA in the form of virus-like replicon particles (VRPs) enables efficient delivery of samRNA to target tissue. The production of these VRPs, however, suffers from contamination with replication-competent virus (RCV) that is thought to arise from recombination events between samRNA and helper RNAs for VRP packaging. The presence of RCV in samRNA in the clinical product is undesirable as alphaviruses may cause serious disease in humans. However, the underlying recombination mechanism leading to RCV is currently unknown. In our work, we demonstrate a detailed evaluation of the recombination sites, which indicates that RCV is formed through an unusual mechanism of “aberrant homologous RNA recombination.” The results are useful for researchers in the field of RNA vaccine manufacture and delivery.

## INTRODUCTION

Synthetic mRNA technology has revolutionized the vaccine field by its rapid deployment in the severe acute respiratory syndrome coronavirus 2 (SARS-CoV-2) pandemic (1–4). In addition to the conventional mRNA vectors, a next-generation of self-amplifying (sa)mRNA vectors is on the rise. These so-called alphavirus “replicon” vectors are based on the positive-stranded alphavirus RNA genome, in which the subgenomic alphavirus structural genes (coding for capsid and envelope glycoproteins) have been substituted for a gene of interest. The replicon-encoded alphavirus non-structural proteins (nsP1–4) amplify the replicon RNA, consequently increasing subgenomic mRNA transcription and thereby expression of the gene of interest. This strategy induces protective humoral and cellular immune responses at lower vaccine doses compared to non-amplifying mRNA vaccines (5, 6). Many types of self-amplifying and novel trans-amplifying mRNAs (7) are being explored (8). Apart from the application as a vaccine platform technology, alphavirus replicon vectors have been engineered for cancer and gene therapy (9).

To efficiently deliver the replicon RNA *in vivo*, it is packaged in virus-like replicon particles (VRPs). These VRPs are made in a production cell line by *in trans* co-expression of the replicon and helper RNA templates, carrying the structural genes flanked by 5′ and 3′UTRs. The resulting VRPs form the clinical product and transduce the replicon RNA to tissues and cells. The replicon RNA cannot spread further than the first cells the VRPs enter, as the VRPs do not contain the helper RNA templates and hence, no alphavirus structural proteins are produced *in vivo*. However, one of the main problems of the VRP production system is the potential for RNA recombination between replicon and helper RNA templates, thereby generating replication-competent viruses (RCVs) (10–14). Contamination of the clinical product with RCV is undesirable as alphaviruses may cause serious disease ranging from chronic arthritis to severe encephalitis (15).

Although RCV formation during VRP production has often been reported, little is known about the conditions required for the occurrence of recombination resulting in RCV. What is clear is that VRP production strategies using mono-helper RNA templates, containing a subgenomic promoter sequence and all alphavirus structural genes on a single RNA template, are typically associated with RCV formation (14, 16, 17). To prevent the formation of RCV, a second generation, so-called “split-helper” VRP production system has been developed, in which the capsid and envelope glycoprotein genes are expressed from two separate helper RNA templates (10–13). The idea is that RCVs are not formed or formed at a very low frequency, because an additional RNA recombination event between the replicon and helper RNA templates is needed to reconstitute a complete viral genome. Finally, a third-generation VRP production system is based on diminishing sequence homology between the replicon and split-helper RNA templates by removal of the subgenomic promoter sequence to further reduce opportunities for RNA recombination (18).

Several studies on replicon helper template designs demonstrated higher recombination frequencies as a result of sequence homology between the replicon and the complementing helper RNA templates (10–12, 17–19). The hypothesis is that this homology can promote a template switch of the RNA-dependent RNA polymerase. The switch point might be located in the homologous sequence itself (homologous RNA recombination), or in a non-shared region near the homologous sequence (a mechanism known as “aberrant homologous RNA recombination”). Aberrant homologous recombination has been described for sequence insertions and deletions, resulting in defective interfering alphavirus genomes (20, 21). In a rare case, RCVs have been generated by illegitimate recombination between non-homologous alphavirus templates (22). Overall, this demonstrates that alphavirus may recombine by (I) homologous, (II) aberrant homologous, or (III) non-homologous recombination. However, as most of the VRP production studies showing RCV formation lack sequence analysis of the RCV recombination site, the exact recombination mechanism resulting in RCVs is currently unknown.

As RCV formation resulting from alphavirus recombination may depend on sequence homology, we experimentally determined RNA recombination outcomes between complementing Venezuelan equine encephalitis virus TC-83 (VEEV) replicon and helper RNA template pairs varying in length of sequences with homology. VEEV replicons were engineered to express capsid or envelope glycoproteins in order to increase sequence similarity with the helper RNA templates. Different combinations of replicon and helper RNA templates with various sequence lengths with homology were co-electroporated in mammalian cells. The culture fluid was serially passaged on Vero cells to evaluate the presence of RCV. The detected RCVs were sequenced to unravel the recombination site and obtain insights in the recombination mechanism. The results increase our fundamental knowledge on the molecular requirements for alphavirus RNA recombination, and assessment of the biosafety of different VRP production systems.

## MATERIALS AND METHODS

### Cells and viruses

Vero cells were cultured at 37°C with 5% CO_2_ in Dulbecco’s modified Eagle medium (DMEM, Gibco) supplemented with 10% fetal bovine serum (FBS, Gibco) and 1% penicillin-streptomycin (Sigma-Aldrich). BHK-21 cells (clone 13, ECACC 85011433) were cultured at 37°C with 5% CO_2_ in DMEM with 5% FBS and 1% penicillin-streptomycin. Venezuelan equine encephalitis virus TC-83 (VEEV, GenBank L01443.1) stocks were generated on Vero cells. Stock titers were determined by end point dilution assays on Vero cells.

### VRP and RCV production by electroporation

Plasmids encoding the VEEV replicon, the capsid-helper, or the envelope-helper RNA were based on the attenuated VEEV TC-83 strain and synthesized by Integrated DNA Technologies. The replicon plasmid carried the non-structural genes, the 26S sub-genomic promoter sequence, and a multiple cloning site in which mCherry, capsid, or envelope genes, or gene fragments were inserted. The two helper constructs contained the first 195 nucleotides of nsP1-encoding region (enhancing replication) and the capsid or envelope genes. The mono-helper was established by insertion of the capsid gene, between the 195 nucleotide residue of the nsP1 encoding region and the start of the E3 gene, in the envelope-helper template. All replicon and helper templates contained the same VEEV TC-83 derived 5′ and 3′UTRs (see also Fig. 2A).

The plasmids encoding the replicon and helper templates were isolated from bacterial cultures by using the NucleoBond Xtra Midi EF purification kit (Macherey-Nagel) and linearized with the NotI restriction enzyme (New England BioLabs). The linearized plasmids were *in vitro*-transcribed (T7 RNA polymerase, New England BioLabs) and capped (Cap structure analog, New England BioLabs) to synthesize capped RNA templates (mRNAs). Various combinations of RNA templates were electroporated into BHK-21 cells. Electroporation was performed using the Gene Pulser Xcell (two pulses of 850 V/25 µF, Bio-Rad) with 0.4 cm cuvettes containing 8 × 10^6^ BHK-21 cells in 800 µL Dulbecco’s phosphate-buffered saline (PBS, Gibco). After electroporation, the cells were resuspended in 10 mL DMEM + HEPES (Gibco) supplemented with 10% FBS and 1% penicillin-streptomycin, and incubated at 37°C with 5% CO_2_. Supernatant fractions were harvested at various time points and analyzed for the presence of VRPs and RCV.

### VRP and RCV titer equivalents

The collected supernatant samples were in duplicate end point diluted on Vero cells to determine VRP and RCV titer equivalents. After overnight incubation, Vero cells expressing the VEEV-nsP2 protein, as a proxy for replicon activity, were stained by immunoperoxidase monolayer assays (IPMA) using goat α-VEEV-nsP2 (1:1,000 in 1% in PBS + 5% FBS, 1 h at room temperature) primary antibody, and rabbit α-goat coupled to alkaline phosphatase (AP) (1:2,500 in PBS + 5% FBS, Sigma, 1 h at room temperature) as secondary antibody. Thereafter, the cells were incubated in AP buffer with NBT/BCIP staining (Roche). The stained nsP2-expressing cells were counted to determine the VRP titer equivalent. The RCV titer equivalent was determined by the virus-induced cytopathic effect (CPE) on Vero cells 5–7 days post-infection.

### Serial passaging assay

Supernatant samples of cells co-electroporated with replicon and helper RNA templates were screened for RCVs by a serial passaging assay in 24-well plates. About 200 µL supernatant sample was added to Vero cell monolayers in 500 µL medium, and incubated for 3–4 days. Thereafter, 200 µL supernatant was passaged to fresh Vero cell monolayers in 500 µL medium, which was repeated for a total of four serial passages, after which cells were screened for CPE. This assay is based on the inability of VRPs to proliferate on Vero cells, whereas RCVs that encode the non-structural and structural proteins can independently form progeny. Accordingly, only RCVs can cause CPE after serial passaging the supernatant on Vero cells. The sensitivity of this detection assay was verified by serial passaging several low concentrations of VEEV TC-83 on Vero cells.

### RT-PCR and Sanger sequencing

RNA was isolated from cells and supernatant of passage 4 samples that showed CPE in the serial passaging assay. The cell and supernatant fractions were homogenized in TRIzol LS reagent (Invitrogen), and RNA was extracted by using chloroform, and precipitated with isopropanol and ethanol according to the manufacturer’s protocol. The purified RNA was dissolved in RNase-free milli-Q water, and reverse transcribed and amplified in a reverse transcriptase-polymerase chain reaction (RT-PCR) by using the SuperScript III one-step RT-PCR system with platinum Taq DNA polymerase (Invitrogen). Specific primers were used that bind to the nucleotide sequences for nsP4 (nsP4 forward primer: 5′-ATGAAACCGTAGGAACTTCCATCA-3′), capsid (capsid forward primer: 5′-ATGTTCCCGTTCCAGCCA-3′, and capsid reverse primer: 5′-CCATTGCTCGCAGTTCTCC-3′), and E3 (E3 reverse primer: 5′-GAGCACATGGGAACGTCAC-3′). The resulting RT-PCR products were visualized on a 1% agarose gel stained with ethidium bromide. RT-PCR products were purified from gel (GFX PCR band and gel band purification kit, Cytiva) and Sanger sequenced (Macrogen). Sequencing results were aligned to replicon, helper, and VEEV reference sequences to determine the RNA recombination sites using Geneious bioinformatics software.

### cDNA library preparation and Nanopore sequencing

Vero cells in T25 flasks were infected with passage 1 supernatants and incubated for 2 days. RNA was isolated from cell flasks using the QIAwave RNA Mini Kit (Qiagen) followed by PolyATtract mRNA Isolation System (Promega) according to the manufacturer’s protocols. The isolated mRNA was reverse transcribed to cDNA using the Maxima H Minus Reverse Transcriptase (ThermoFisher) following the Ligation sequencing V14 - Direct cDNA sequencing (SQK-LSK114) protocol and cDNA sequencing libraries were prepared using the SQK-LSK114 ligation kit (Oxford Nanopore Technologies). The libraries (5–15 ng) were loaded onto MinION R10.4.1 flow cells and sequenced using a MinION Mk1C sequencer (Oxford Nanopore Technologies). Raw reads were basecalled in SUP-mode using Dorado v0.5.3, and reads with a *q-*score above 10 were exported as .fastq-files. Resulting Fastq files were filtered on read lengths greater than 2 kb. These large reads were mapped to VEEV replicon, capsid, or envelope reference sequences using Geneious bioinformatics software with the additional command line -Y. The extracted read lists were cleared from secondary alignments that aligned in multiple directions or locations to the reference sequence. The resulting read lists were combined to find duplicate reads in the combined lists that represented recombinant reads. Reads were manually inspected for recombination sites.

## RESULTS

### Design of an RCV detection assay

**Fig 1:**
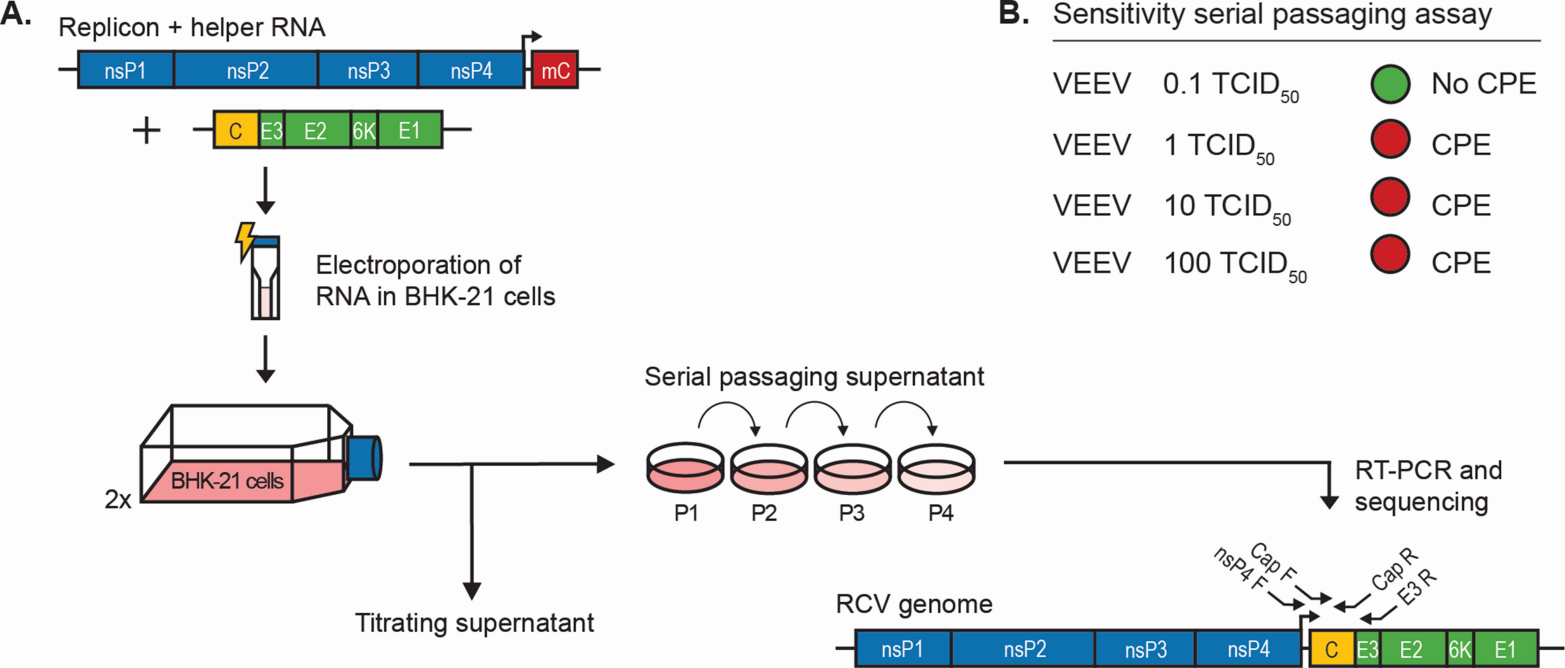
Schematic representation of the RCV detection assay. (**A**) Replicon and helper RNA templates were electroporated in BHK-21 cells, which were then divided over two T25 cell culture flasks. At 24, 48, and 72 hpe, supernatant samples were taken for titrations and serial passaging. Titrations by end point dilution assay were scored on CPE and nsP2 expression, resulting in the RCV and VRP titer equivalents, respectively. Supernatant samples were serial passaged for a total of four passages on Vero cells. Detection of CPE after serial passage four indicated the potential presence of an RCV. To confirm RCV presence, RNA was purified from passaged medium fractions that showed clear CPE, and RT-PCR was performed using VEEV-specific nsP4 forward (nsP4 F), capsid forward (cap F), capsid reverse (cap R), and E3 reverse (E3 R) primer sets. RT-PCR confirmed that RCVs were sequenced to investigate the recombination junction site. (**B**) Sensitivity of the serial passaging assay to detect RCVs. Vero cells were infected with 0.1–100 TCID_50_ units VEEV per 1 × 10^5^ cells. Supernatant fractions were passaged on fresh Vero cells for a total of four serial passages. Detection of virus infection by clear CPE is indicated in red (*n* = 2).

To investigate how sequence homology orchestrates RNA recombination, complementing VEEV replicon and helper RNA templates varying in length of sequences with homology were co-electroporated into BHK-21 cells (Fig. 1A). The electroporated cells were divided over two culture flasks, generating a duplicate for easier evaluation of independent recombination events. At 24, 48, and 72 h post-electroporation (hpe), supernatant samples were collected to evaluate VRP production and potentially RCV generation. First of all, the supernatant samples were titrated in an end point dilution assay to determine the VRP and RCV titers. We generally quantify VRPs by immunoperoxidase staining of nsP2-expressing cells in an end point dilution assay. However, as in this case, both VRPs and RCVs would express nsP2, this quantification was here termed the VRP titer equivalent (as only limited amounts of RCV were expected). Similar, RCV titers are generally quantified by CPE detection in an end point dilution assay (RCV titer equivalent), despite the fact that VRPs also induced some level of CPE in the lower dilutions of the end point dilution assay.

Next, the collected supernatant samples were serially passaged on Vero cells to further evaluate the presence of RCVs. Only an RCV encoding all non-structural and structural proteins is able to replicate and proliferate when serially passaged on cells, whereas VRPs (encapsulated replicon RNA) cannot proliferate as they do not encode (all) structural genes. This cell-based serial passaging assay was shown to be sensitive, as an initial infection of 1 TCID_50_ unit of VEEV per 1 × 10^5^ cells was detectable by clear CPE within four serial passages on Vero cells (Fig. 1B). Accordingly, it was expected that a low concentration of RCVs would be detectable as well. To confirm RCV formation resulting from RNA recombination between replicon and helper RNA templates, the supernatants of cells that showed clear CPE were subjected to a one-step RT-PCR using three specific VEEV primer sets that spanned the expected recombination site. Finally, the recombination site was sequenced to identify the recombination mechanism.

### Co-expression of VEEV replicon and helper RNA templates results in VRPs and putative RCV

**Fig 2:**
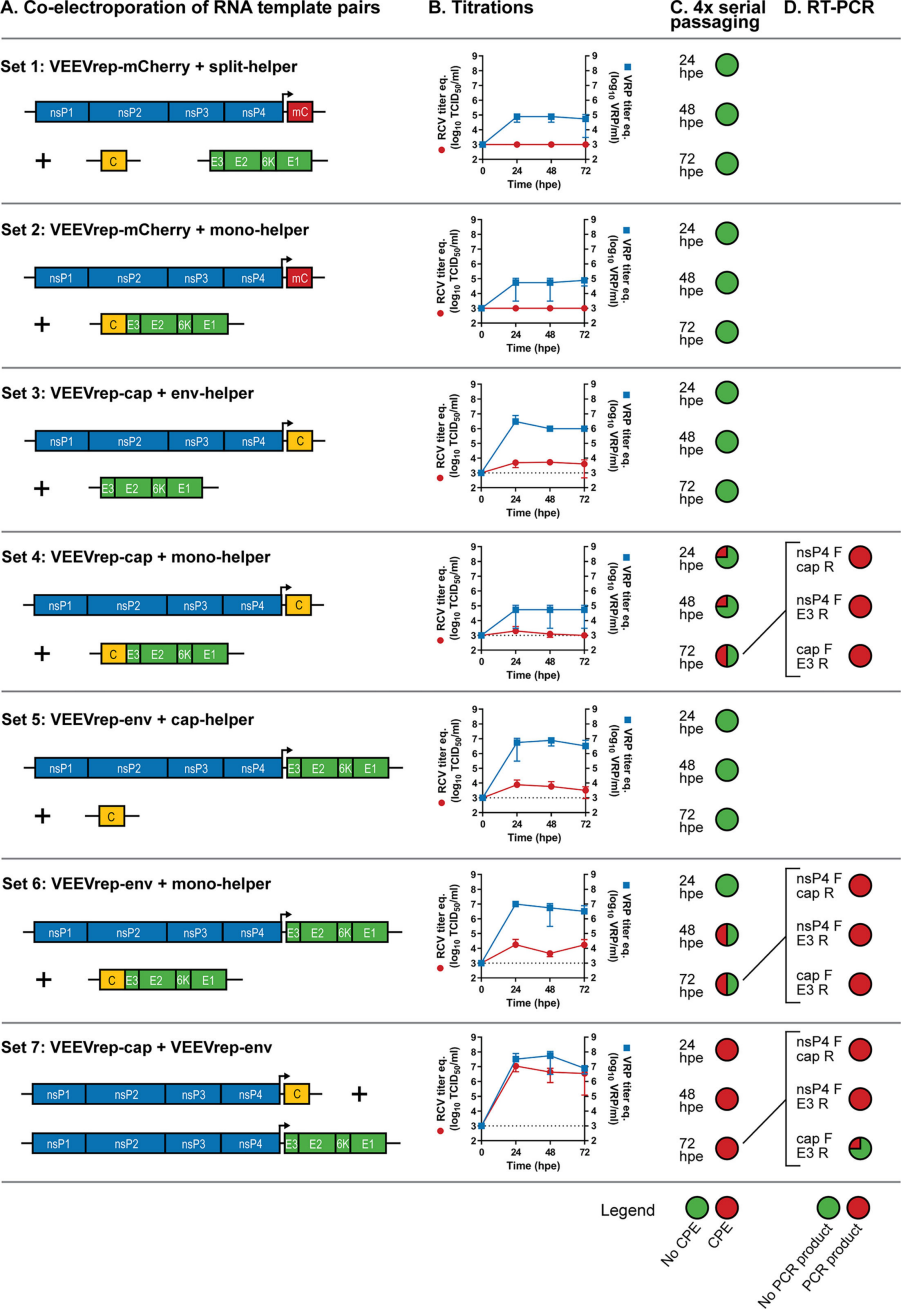
Evaluating recombination between complementing VEEV RNA template pairs varying in length of sequence homology. (**A**) VEEV replicon and helper RNA template pairs varying in length of sequence homology were co-electroporated in BHK-21 cells in duplicate. The VEEV replicon templates encoded the VEEV non-structural proteins (nsP1–4), the 26S subgenomic promoter (arrow), and either mCherry (VEEVrep-mCherry), capsid (VEEVrep-cap), or envelope glycoproteins (VEEVrep-env). The VEEV helper templates encoded either the capsid (cap-helper), envelope glycoproteins (env-helper), or the complete structural cassette (mono-helper). All RNA templates contained a capped 5′UTR and polyadenylated 3′UTR (black lines). (**B**) The RCV and VRP titer equivalents (eq.) were determined by end point dilution assay (detection limit 1 × 10^3^ TCID_50_/mL and 1 × 10^3^ VRPs/mL) at indicated time points scored on, respectively, induced CPE and expression of nsP2. Error bars indicate standard deviation of the mean (*n* = 4). (**C**) Serial passaged supernatant samples collected at indicated time points post-electroporation were monitored for RCV presence by CPE. Detection of CPE after serial passaging is indicated in red as percentage in circular chart (*n* = 4). (**D**) Potential RCVs detected by CPE in passage 4 of the serial passaging assay were analyzed by RT-PCR. RNA was purified from passaged 72 hpe samples. Detection of viral sequences by PCR products using the VEEV-specific nsP4 forward and capsid reverse (nsP4 F + cap R), nsP4 forward and E3 reverse (nsP4 F + E3 R), and capsid forward and E3 reverse (cap F + E3 R) primer sets are indicated in red as percentage in circular chart (VEEVrep-cap + mono-helper *n* = 2, VEEVrep-env + mono-helper *n* = 2, VEEVrep cap + VEEVrep env *n* = 4).

Complementing replicon and helper RNA template pairs were generated based on the VEEV genome to evaluate the influence of sequence homology on RNA recombination (Fig. 2A). The VEEV replicon RNA templates encoded the VEEV non-structural proteins (nsP1–4), the 26S subgenomic promoter, and either mCherry (VEEVrep-mCherry), capsid (VEEVrep-cap), or envelope glycoproteins (VEEVrep-env). The VEEV helper RNA templates encoded either the capsid (cap-helper), envelope glycoproteins (env-helper), or the complete structural cassette (mono-helper). Various combinations of complementing templates with and without sequence homology were co-electroporated into BHK-21 cells in duplicate. As the electroporated cells in each electroporation cuvette were divided over two culture flasks (Fig. 1), a total of four replicates per template pair were evaluated for VRP and RCV production.

The VRP and RCV titer equivalents were determined from supernatant samples collected at 24, 48, and 72 hpe (Fig. 2B). This showed that maximum VRP production was established within 24 h with VRP titer equivalents reaching values between 10^5^ and 10^8^ VRP/mL. Thereafter, the levels stagnated or slightly decreased as cell death was induced by continued expression of viral proteins from the replicon. The template pairs that reached titers of >10^6^ VRP/mL (VEEVrep-cap and env-helper, VEEVrep-env and cap-helper, VEEVrep-env and mono-helper, and VEEVrep-cap and VEEVrep-env) also showed some CPE in the end point dilution assay titrations, indicative of potential low levels of RCV. Interestingly, the VEEVrep-cap and VEEVrep-env template combination generated RCV titer equivalents, which were comparable to the VRP titer equivalents. This implied that either the VEEVrep-cap and VEEVrep-env templates were efficiently spreading simultaneously as complementing VRPs in a “multipartite virus” mechanism, or that efficient RNA recombination indeed resulted in high titers of RCV causing clear CPE in the higher dilutions of the end point dilution assay.

### Recombination between complementing VEEV RNA template pairs results in RCV formation

To further assess RNA recombination between complementing VEEV replicon and helper RNA templates, the supernatant samples of co-electroporated cells collected at 24, 48, and 72 hpe were serially passaged on Vero cells (Fig. 2C). After four serial passages, clear CPE was observed for the co-electroporated VEEVrep-cap and mono-helper, VEEVrep-env and mono-helper, and VEEVrep-cap and VEEVrep-env template pairs. This suggested the presence of RCV as a result of recombination between VEEV replicon and helper RNA templates, and between the two VEEV replicon RNA templates. To confirm the presence of RCVs, RNA was extracted from the samples that showed clear CPE, and RT-PCR was performed using three specific VEEV primer sets (Fig. 2D). The existence of RCV could only be confirmed if all three primer sets (nsP4 forward and cap reverse, nsP4 forward and E3 reverse, and cap forward and E3 reverse) would generate a PCR product (see Fig. 1A).

The RT-PCR analysis confirmed that half of the VEEVrep-cap and mono-helper co-electroporated replicates resulted in RCV. The two replicates that caused CPE in the serial passaging assay (Fig. 2C, set 4), also resulted in PCR products with all three primer sets (Fig. 2D, set 4). These resulted from independent duplicates of the electroporation, suggesting that these are independent recombination events. One of the two RCVs was already generated in the first 24 hpe and persisted over time, whereas the second RCV was only detected at 72 hpe. Recombination between the VEEVrep-env and mono-helper was also confirmed (Fig. 2A, set 6). The two samples that caused CPE in the serial passaging assay (Fig. 2B, set 6), showed PCR products for all three primer sets (Fig. 2C, set 6). Both RCVs developed between 24 and 48 hpe and persisted over time. They originated from the same electroporation. Finally, RNA recombination was confirmed between the VEEVrep-cap and VEEVrep-env templates (Fig. 2A, set 7). Although all four independent VEEVrep-cap and VEEVrep-env co-electroporation caused CPE in the serial passaging assay for samples harvested at 24, 48, and 74 hpe (Fig. 2C, set 7), only one RCV was confirmed by RT-PCR analysis using the cap forward and E3 reverse primer set (Fig. 2D, set 7). It was evaluated if an RCV was developed in the order of “nsP4-env-cap” by an extended extension time of the nsP4 forward and cap reverse RT-PCR reaction, but this did not result in a product with the expected length for such RCV. So, the other three VEEVrep-cap and VEEVrep-env template replicates apparently generated high concentrations of VRPs, which efficiently complemented each other, and thereby caused CPE upon serial passaging over several passages.

Overall, from all combinations tested, RCVs were only formed upon co-electroporation of complementing RNA template pairs sharing identical sequences, suggesting that RCV formation was the result of homologous or aberrant homologous RNA recombination.

### RCVs were generated by aberrant homologous RNA recombination

**Fig 3:**
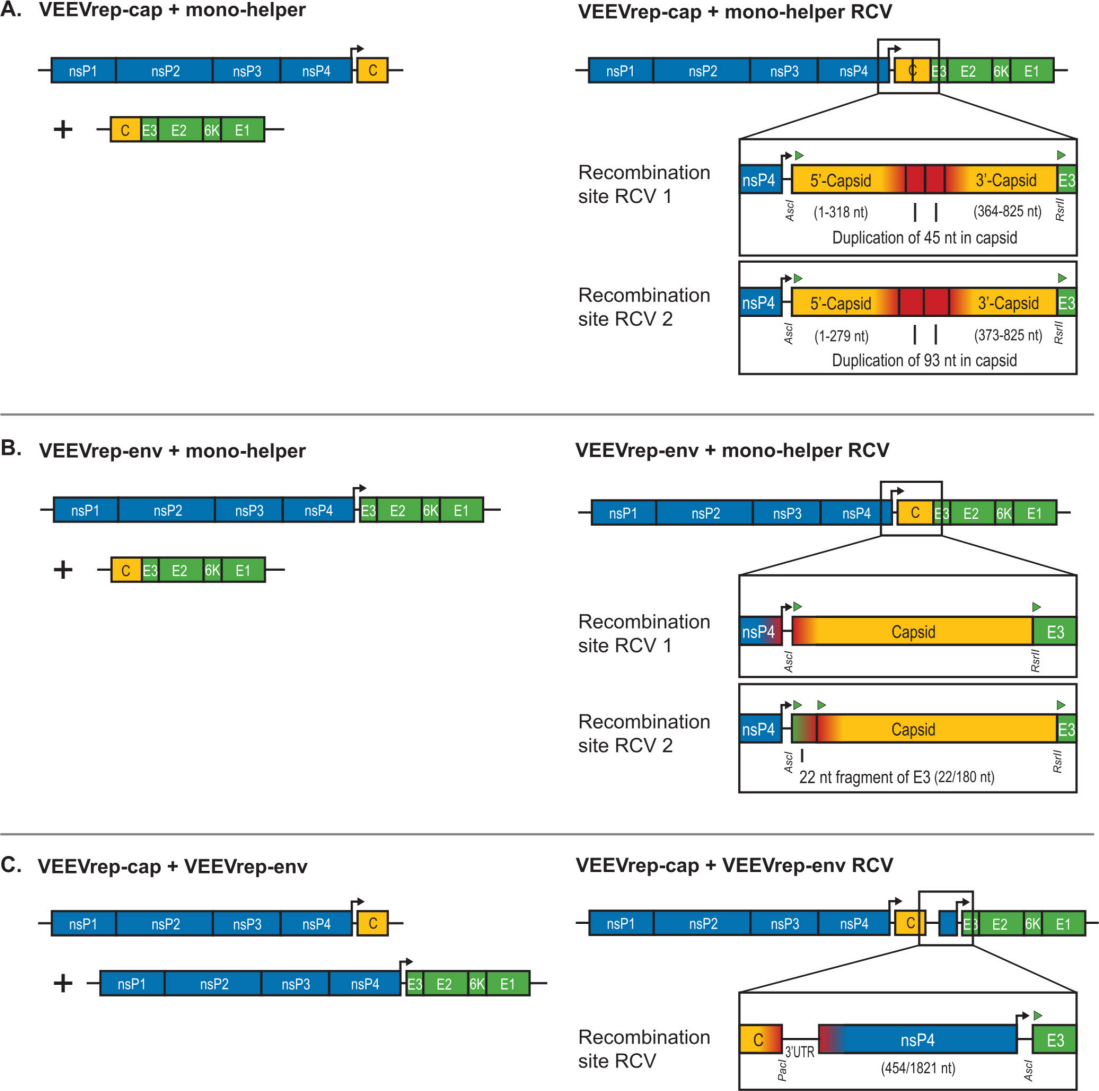
Genome composition of detected RCVs. RT-PCR products of nsP4 F and cap R, nsP4 F and E3 R, and cap F and E3 R primer set amplifications were purified and Sanger sequenced to verify the recombination sites. (**A**) The VEEVrep-cap and mono-helper templates recombined in the homologous capsid sequence, resulting in two similar RCVs with sequence duplications of 45 and 93 nt (indicated in red). (**B**) The VEEVrep-env and mono-helper templates with homology in the envelope gene sequence recombined between the start of the VEEVrep-env’s E3 sequence and the start of the mono-helper (indicated in red), resulting in two slightly different RCVs; without and with a 22-nt fragment of the 180 nt E3 gene. (**C**) The VEEVrep-cap and VEEVrep-env templates recombined between the VEEVrep-cap 3′UTR and VEEVrep-env nsP4 (indicated in red). The RCV encoded thereby an additional 454 nt of the 1,821 nt nsP4 gene. VEEV replicon and helper template-specific restriction sites are indicated by restriction enzyme names, and start of open reading frames are indicated by green triangles.

The RCVs were sequenced to identify the recombination site and possibly unravel the template switch mechanism (Fig. 3). Sanger sequencing of RT-PCR products demonstrated that all RCVs were established by a single template switch. In several RCVs, we found clear traces of this particular template switch (e.g., duplications of sequence elements), and although this was not the case in all RCVs, they were all distinguishable from potential wild-type VEEV contamination. In contrast to wild-type VEEV, the VEEV replicon templates contained an additional restriction site in the junction UTR between nsP4 and the gene of interest, and the mono-helper template contained an additional restriction site between the capsid and envelope genes (Fig. 3). At least one of the specific replicon and helper sequence elements was detected in each RCV. Moreover, the sequencing results demonstrated a similarity in recombination site, and hence underlying mechanism, of RCVs that arose from the same template pairs, which is explained below.

The two individual VEEVrep-cap and mono-helper RCVs showed a recombination site roughly halfway through the capsid gene with in-frame sequence duplications of 45 and 93 nucleotides, which are not found in the wild-type sequence (Fig. 3A). These duplications of amino acids 106–121 and 93–124 correspond to the linker sequence between the N- and C-terminal domain (109–125 aa) of the capsid protein creating a repetition of the ribosomal binding site (105–116 aa) and an incomplete duplication of the packaging signal region (111–126 aa) (23). The sequence duplications indicated that homology played a role in recombination, and since the template switch occurred near this homologous region, it was concluded that the VEEVrep-cap and mono-helper recombined via aberrant homologous recombination.

The two RCVs resulting from VEEVrep-env and mono-helper template combination showed both template switches at the start of VEEVrep-env’s E3 sequence which was connected to the start of the mono-helper’s capsid sequence (Fig. 3B). This caused the deletion of the VEEVrep-env’s structural genes, except for a 22 nucleotide fragment of E3 in the second RCV. In this 22 nucleotides E3 residue a micro-homology sequence was observed of 7 nucleotides overlapping with the start of the capsid ORF, including its start codon, of the mono-helper. Nevertheless, as this overlap was so small and larger homology stretches were observed outside the recombination site, we consider this as aberrant homologous recombination.

The VEEVrep-cap and VEEVrep-env RCV also demonstrated aberrant homologous recombination. The VEEV replicon templates were homologous in the nsP1–4 region, but the template switch occurred between the VEEVrep-cap 3′UTR and the VEEVrep-env nsP4 gene, which had no homologous sequence overlap (Fig. 3C). In this recombination event, the 454 nucleotides 5′-end of VEEVrep-env nsP4 provided a second 26S subgenomic promoter to the RCV, which allowed separated subgenomic RNA synthesis of the capsid and envelope genes.

### Early detection of RCVs using Nanopore long-read sequencing

**Fig 4:**
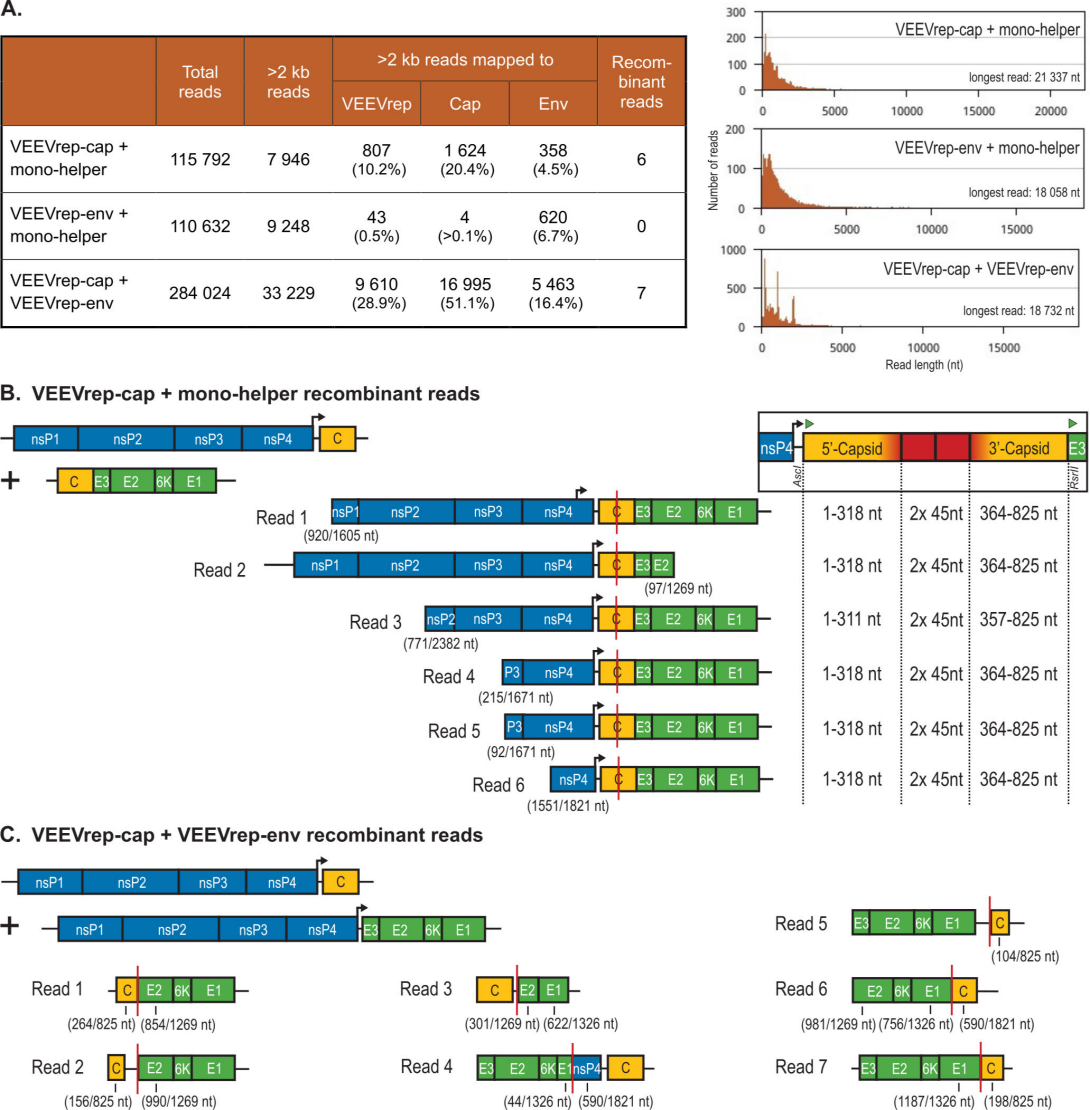
Genome composition of detected recombinant Nanopore reads. (**A**) Cell fractions extracted from the first supernatant passage were Nanopore long-read RNA sequenced. To allow identification of reads with clear recombination sites, only reads longer than 2 kb were considered for analyses. These long reads were mapped to VEEV replicon (VEEVrep), capsid (cap), and envelope (env) reference sequences. Reads that aligned to both the VEEVrep and env sequence, VEEVrep and cap sequence, or the cap and env sequence for respectively VEEVrep-cap and mono-helper, VEEVrep-env and mono-helper, and VEEVrep-cap and VEEVrep-env combinations, were considered recombinant reads if both alignments were detected in one orientation of at least 2 kb. Identified recombinant reads are illustrated for (**B**) the VEEVrep-cap and mono-helper templates, and (**C**) the VEEVrep-cap and VEEVrep-env templates. Genes of which only fragments were annotated are indicated with length of the gene fragment relative to the total gene length. Recombination sites are indicated by red vertical lines. (**B**) The six recombinant reads of the VEEVrep-cap and mono-helper template combination have recombination sites in the homologous capsid sequence with sequence duplications of 45 nt. (**C**) The seven recombinant reads of the VEEVrep-cap and VEEVrep-env template varied in capsid and envelope order.

The identified RCVs (Fig. 3) were isolated after four cellular passages which might have accommodated changes to the original RCV sequence. To determine the RCV sequences at an earlier stage, total RNA was isolated from passage 1 and sequenced using the Nanopore long-read sequencing technology. In total, three samples were sequenced: the VEEVrep-cap and mono-helper combination that resulted in RCV 1 (Fig. 3A), the VEEVrep-env and mono-helper combination that resulted in RCV 2 (Fig. 3B), and the VEEVrep-cap and VEEVrep-env combination that resulted in a RCV (Fig. 3C). The resulting long reads were aligned to VEEV replicon, capsid, and envelope reference sequences (Fig. 4A). This demonstrated variation in aligned reads per sample. The VEEVrep-cap and VEEVrep-env combination resulted in more than two times the total amount of reads and the highest percentages of reads that mapped to the VEEV replicon, capsid, and envelope reference sequences (Fig. 4A). This correlated with the higher RCV titer compared to the VEEVrep and mono-helper combinations (Fig. 2).

The VEEVrep-cap and mono-helper sample demonstrated more reads that mapped to the VEEV replicon, capsid, and envelope reference sequences than the VEEVrep-env and mono-helper sample. Of these mapped reads, the majority represented, as expected, the replicon or the subgenomic expressed capsid or envelope gene. For the VEEVrep-cap and mono-helper sample also an abundant amount of reads mapped to the envelope reference sequence, potentially representing the generated RCV. Six of these reads were identified as recombinant reads, aligning to VEEV replicon (non-structural), capsid, and envelope sequences (Fig. 4B). Interestingly, from the six recombinant reads five showed an identical recombination site as the previously identified VEEVrep-cap and mono-helper RCV 1 that was detected after four passages (Fig. 3A). In contrast, none of the VEEVrep-env and mono-helper reads were identified as recombinant reads, whereas an RCV was detected in this sample after four passages. Based on the low number of aligned reads, we hypothesize that this RCV was not abundant enough in passage 1 to be detected as a recombinant read using Nanopore sequencing.

The reads from the VEEVrep-cap and VEEVrep-env combination were also examined for recombination patterns. Although seven recombinant reads were identified (Fig. 4C), none of these represented the previously detected RCV (Fig. 3C) or a clear RCV in general. These Nanopore reads all demonstrated recombination between capsid and envelope genes, resulting in fragmented capsid and envelope genes. The recombinant reads were too short to evaluate if these reads represented viable RCV, and could potentially be artifacts of random template switching during the RNA seq library preparation (24). Although the Nanopore long read sequencing resulted in a relevant RCV overview for the VEEVrep-cap and mono-helper combination in passage 1, this method was not beneficial in the early detection of expected RCVs for the other two samples.

### Short sequence overlaps promote recombination

**Fig 5:**
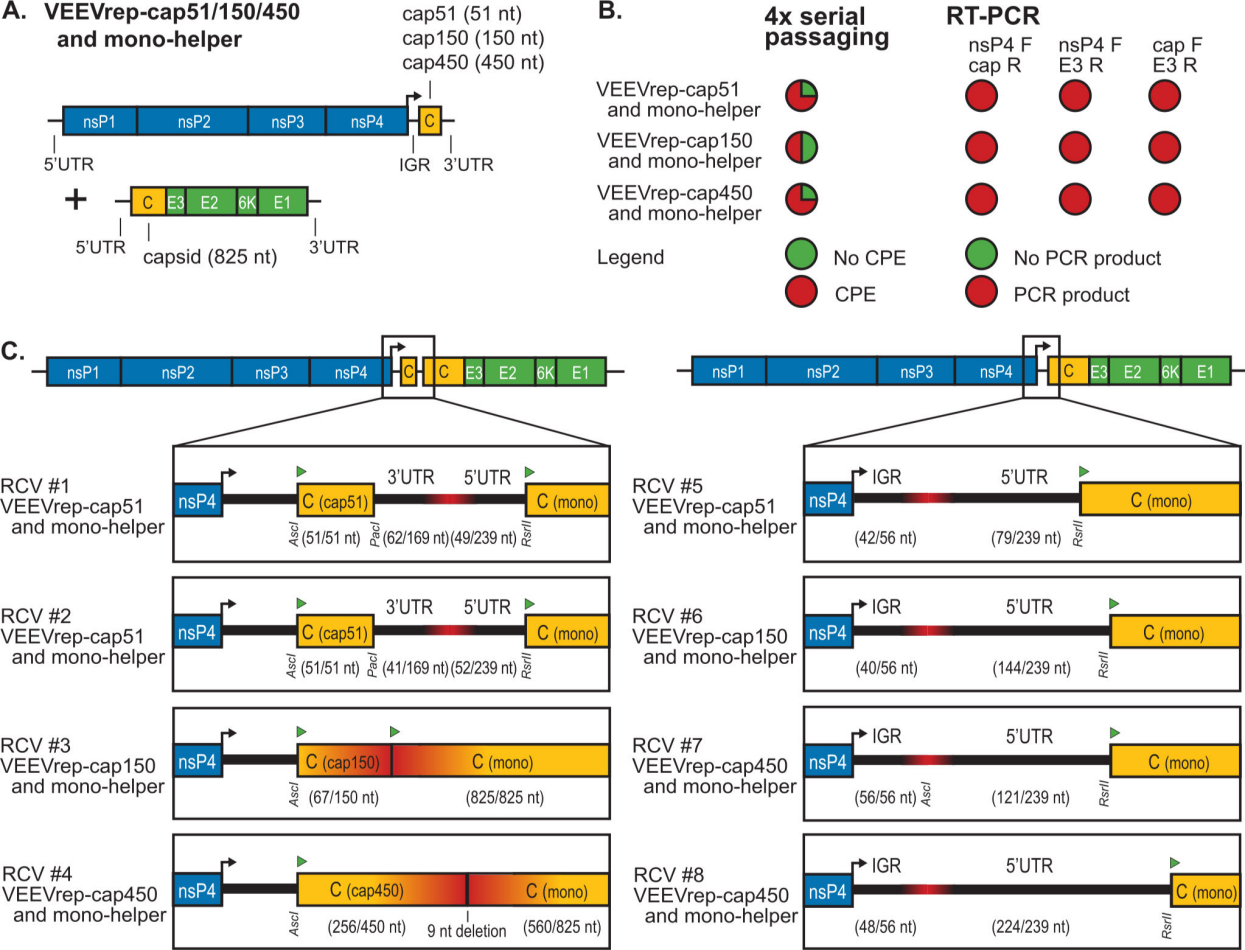
Recombination between complementing VEEV RNA template pairs with minimal length of sequence homology. (**A**) VEEV replicon templates carrying capsid gene fragments were co-electroporated in BHK-21 cells with mono-helper RNA. The VEEV replicon templates contained the VEEV non-structural protein (nsP1–4) genes, the 26S subgenomic promoter (arrow), the intergenic region (IGR), and either the first 51 nt (VEEVrep-cap51), 150 nt (VEEVrep-cap150), or 450 nt (VEEVrep-450) of the capsid sequence. The VEEV mono-helper template encoded the complete structural cassette. All RNA templates contained a capped 5′UTR and polyadenylated 3′UTR (black lines). (**B**) Serial passaged supernatant samples collected at 72 h post-electroporation were monitored for RCV presence by CPE. Detection of CPE after serial passaging is indicated in red as percentage in circular chart (*n* = 4). Potential RCVs detected by CPE in passage 4 of the serial passaging assay were analyzed by RT-PCR. RNA was purified from passaged 72 hpe samples. Detection of viral sequences by PCR products using the VEEV-specific nsP4 forward and capsid reverse (nsP4 F + cap R), nsP4 forward and E3 reverse (nsP4 F + E3 R), and capsid forward and E3 reverse (cap F + E3 R) primer sets is indicated in red as percentage in circular chart (VEEVrep-cap51 + mono-helper *n* = 3, VEEVrep-cap150 + mono-helper *n* = 2, VEEVrep-cap450 + mono-helper *n* = 3). (**C**) RT-PCR products of nsP4 F and E3 R primer set amplifications were purified and Sanger sequenced to verify the recombination sites. The recombination junctions are indicated in red, VEEV replicon and helper template specific restriction sites are indicated by restriction enzyme names, and start of open reading frames are indicated by green triangles.

From the tested sets of complementing RNA template pairs, RCVs were only formed upon co-electroporation of template pairs sharing an identical gene sequence of which the shortest, the capsid-encoding sequence, was 825 nt in length. To evaluate the influence of the length of the identical sequences on aberrant homologous recombination, three additional replicon templates were generated carrying the first 51, 150, or 450 nt of the capsid-encoding region (Fig. 5A). These templates were co-electroporated with a complementing mono-helper, and evaluated on RCV formation using the previously described serial passaging assay, followed by RT-PCR and sequencing (Fig. 1).

Interestingly, even the shortest homologous sequence of 51 nt in the VEEVrep-cap51 assisted RCV formation with the complementing mono-helper. Three out of four co-electroporated VEEVrep-cap51 and mono-helper replicates resulted in RCV, which were detected by CPE in the serial passaging assay and confirmed by RT-PCR (Fig. 5B). Furthermore, two VEEVrep-cap150 and mono-helper RCVs, and three VEEVrep-cap450 and mono-helper RCVs were isolated (Fig. 5B).

Sanger sequencing of the recombination junctions demonstrated that template switching had occurred near the short homologous capsid sequence fragments, but not in all RCVs, 51–450 nt capsid sequence fragment of the VEEV replicon was retained (Fig. 5C). Two RCVs carried the complete 51 nt capsid fragment of VEEVrep-cap51 (Fig. 5C #1 and #2), one RCV had a 66 nt duplication of the capsid fragment from VEEVrep-cap150 (Fig. 5C #3), and in one RCV the first 256 nt of the capsid from VEEVrep-cap450 were fused with 560 nt of the capsid from the mono-helper (Fig. 5C #4). The later RCV showed a 9 nt deletion in the recombination junction, deleting three amino acids of the third subdomain of the N-terminal capsid region which contains the nuclear localization signal (23). In the other RCVs, the VEEV replicon’s capsid sequence was not retained and showed a template switch between the replicon’s intergenic region (IGR) and mono-helper’s 5′UTR (Fig. 5C #5 to #9). The length of the IGR and the 5′UTR varied between the RCVs; from 41 to 56 nt of the IGR (56 nt total) and from 78 to 224 nt of the 5′UTR (total 239 nt).

Overall, this experiment demonstrated that homologous sequence overlap of 51 nt between the replicon’s subgenomic region and mono-helper is sufficient to promote aberrant homologous RNA recombination (Fig. 5). The location of the overlap is important as homologous overlap in replicon and helper’s 5′ and 3′UTRs of, 229 and 169 nt, respectively, did not result in RCVs after co-electroporating VEEVrep-mCherry and mono-helper (Fig. 2).

## DISCUSSION

To better understand the conditions that facilitate RCV formation during VRP manufacturing, the influence of sequence homology between replicon and helper template pairs on alphavirus RNA recombination was evaluated. Complementing VEEV replicon and helper templates with or without homologous sequence overlap were co-expressed in mammalian cells to produce VRPs. The culture fluid with VRPs was serially passaged to evaluate the presence of RCVs based on the observation of CPE. This assay was a sensitive method as a minimal concentration of 1 TCID_50_ unit VEEV was detectable by clear CPE within four serial passages on Vero cells. However, a limitation of this method was the inability to select for RCVs in the presence of a “multipartite virus” mechanism in case of the VEEVrep-cap and VEEVrep-env template pairs, which also resulted in CPE in the serial passaging assay irrespective of the presence of RCVs. This was solved by an additional analysis based on amplification of the expected recombination site via trifold RT-PCR analysis. Combining the results of the serial passaging assay of various replicon and RNA template combinations with the RT-PCR data enabled detection of 13 different RCVs, which were the result of recombination of the combinations VEEVrep-cap and mono-helper, VEEVrep-env and mono-helper, VEEVrep-cap and VEEVrep-env, VEEVrep-cap51 and mono-helper, VEEVrep-cap-150 and mono-helper, and VEEVrep-cap450 and mono-helper, respectively. Probably only a couple of cells initially secreted these RCVs as the corresponding RCV titer equivalents were low, indicating that the RCVs made up only a small fraction of the total encapsulated genome population (VRP + RCV). This made the early detection of RCVs by Nanopore long-read sequencing in passage 1 challenging.

Based on the detected RCVs, we conclude that RNA recombination between the various VEEV templates is homology-assisted, because RCVs were only formed upon co-electroporation of RNA templates with a sequence homology in the non-structural or structural genes. Notably, sequence overlap was not detected at the recombination junction sites, indicating that RCV formation is a result of aberrant homologous RNA recombination. The overlap in genes or gene fragments likely triggered the recombination event to occur. We initially hypothesized that this overlap created some latitude for recombination as it potentially prevents truncation of essential genes during a non-homologous recombination event. Truncation of essential genes would result in defective viral genomes that cannot proliferate independently and would not be detected as RCV. However, as the mCherry gene in VEEVrep-mCherry created a similar latitude as the envelope gene in VEEVrep-env, but did not generate RCVs with the mono-helper, this hypothesis was dismissed. Moreover, the location of the sequence overlap between replicon and helper is important. Whereas 51 nt sequence homology in the non-structural genes was enough to promote RCV formation, 169 nt overlap in the 3′UTRs and 229 nt overlap in the 5′UTRs of replicon and helper did not result in RCVs. Accordingly, we conclude that sequence homology in the non-structural and structural genes promotes, or may be even required for, the VEEV RNA recombination process to generate RCVs, but that the actual event may occur in a non-homologous section.

The exact molecular mechanism behind the observed aberrant homologous recombination process is still not fully clear. Possibly, the recombination events between overlapping RNAs may occur in the limited time window between negative-strand RNA synthesis and spherule formation, at a time when dsRNA intermediates are formed in the cytoplasm but are not yet fully protected by a membrane. Alternatively, two adjacent spherules with different, replicating dsRNA molecules may fuse, providing opportunity for recombination. These events are likely rare and hard to detect, although RCV coming out as a result will spread and this facilitates detection by Nanopore sequencing or RT-PCR.

In this study, RCVs were generated between templates with complete sequence homology in either the non-structural protein, capsid, or envelope genes. However, smaller homologous fragments or conserved sequence elements might already assist alphavirus recombination, for example, homology in the conserved nsP4 gene. If the conserved sequences in nsP4 facilitate (aberrant) homologous recombination, this could explain the historical recombination event between the distantly related member of the Sindbis virus and eastern equine encephalitis virus lineage resulting in western equine encephalitis virus (WEEV), the only known natural alphavirus chimera. Unfortunately, it is hard to trace back the homology between the ancestors and the exact recombination site as WEEV was established at least 1600 years ago, allowing multiple mutations in the once ancestral viruses and WEEV itself over the years (25).

Positional analysis of template switching events suggests the existence of specific RNA recombination hotspots in a study on the formation of defective interfering alphavirus RNA genomes. Genome deletions were frequently found in between the nsP4 and E1 gene, and in between the capsid and E1 gene (20, 24, 26). In line with this, we observed recombination sites between the VEEVrep-env and mono-helper in the very same position. Although a similar recombination site was expected for the VEEVrep-cap and mono-helper, the recombination site was observed in the middle of the capsid gene. The difference in recombination sites between the VEEVrep-cap and VEEVrep-env in their interaction with the mono-helper could be a result of (I) homology-assisted recombination, (II) secondary RNA structures that cause RdRp stutter and re-attach, or (III) better survival chances of the recombinant when gene functionality was not disrupted. The VEEVrep-cap and VEEVrep-env mutually recombined in a different location; between the 3′-UTR and nsP4 gene. This recombination event resulted in an RCV with duplicated 26S subgenomic promoters that separately supported the expression of the capsid and envelope genes. A similar RCV with a duplication of the 26S promoter has been described before for a recombination event between the VEEV replicon and helper RNA templates (18).

The fact that no RCV formation was observed between the VEEV replicon and helper templates that lack sequence homology in non-structural and structural genes supports the safe application of the present mono- and split-helper systems for VRP production, in contrast to the conventional mono-helper design. The conventional mono-helper contained the 26S subgenomic promoter that directs the production of the structural genes. As this 26S promoter is also encoded in the replicon RNA to drive amplification of the gene of interest, the conventional 26S-based mono-helper frequently generated RCVs by homologous recombination. However, since the 26S promoter is not required on the VEEV helper RNA templates to produce VRPs, promoterless VEEV helpers were designed (18). Here, we show that VRPs can safely be produced using a promoterless mono-helper system providing that the VEEV replicon does not encode any other homologous structural genes or parts thereof. The application for promoterless mono-helper system can therefore be applied in VRP production to reduce *in vitro*-RNA transcription costs, nevertheless the promoterless split-helper system will still be the safest VRP production option with comparable VRP production efficiency.

In all, this study provides evidence that alphavirus RNA recombination resulting in RCVs is homology-assisted. Recombination events leading to RCV formation were detected between complementing VEEV replicon and helper template pairs with sequence homology, but not between non-homologous template pairs. The lack of viable recombination events between non-homologous sequences underlines the safety of the latest VEEV replicon-helper vaccine platforms in terms of absence of RCV generation in the VRP production process.
